# Polysubstance use poisoning deaths in Canada: an analysis of trends from 2014 to 2017 using mortality data

**DOI:** 10.1186/s12889-022-12678-z

**Published:** 2022-02-10

**Authors:** Sarah Konefal, Adam Sherk, Bridget Maloney-Hall, Matthew Young, Pam Kent, Emily Biggar

**Affiliations:** 1 Canadian Centre on Substance Use and Addiction, Ottawa, Ontario Canada; 2 Canadian Institute for Substance Use Research, Victoria, B.C. Canada; 3 Gambling Research Exchange Ontario, Guelph, Ontario Canada; 4 Department of Psychology, Carleton University , Ottawa, Ontario Canada

**Keywords:** Polysubstance mortality, Opioid poisoning, Stimulants, Polysubstance use, Intentional poisoning, Unintentional poisoning

## Abstract

**Background:**

Over the past decade, rates of drug poisoning deaths have increased dramatically in Canada. Current evidence suggests that the non-medical use of synthetic opioids, stimulants and patterns of polysubstance use are major factors contributing to this increase.

**Methods:**

Counts of substance poisoning deaths involving alcohol, opioids, other central nervous system (CNS) depressants, cocaine, and CNS stimulants excluding cocaine, were acquired from the Canadian Vital Statistics Death Database (CVSD) for the years 2014 to 2017. We used joinpoint regression analysis and the Cochrane-Armitage trend test for proportions to examine changes over time in crude mortality rates and proportions of poisoning deaths involving more than one substance.

**Results:**

Between 2014 and 2017, the rate of substance poisoning deaths in Canada almost doubled from 6.4 to 11.5 deaths per 100,000 population (Average Annual Percent Change, AAPC: 23%, p < 0.05). Our analysis shows this was due to increased unintentional poisoning deaths (AAPC: 26.6%, p < 0.05) and polysubstance deaths (AAPC: 23.0%, p < 0.05). The proportion of unintentional poisoning deaths involving polysubstance use increased significantly from 38% to 58% among males (p < 0.0001) and 40% to 55% among females (p < 0.0001). Polysubstance use poisonings involving opioids and CNS stimulants (excluding cocaine) increased substantially during the study period (males AAPC: 133.1%, p < 0.01; females AAPC: 118.1%, p < 0.05).

**Conclusions:**

Increases in substance-related poisoning deaths between 2014 and 2017 were associated with polysubstance use. Increased co-use of stimulants with opioids is a key factor contributing to the epidemic of opioid deaths in Canada.

**Supplementary Information:**

The online version contains supplementary material available at 10.1186/s12889-022-12678-z.

## Background

It is estimated that over 27,000 deaths were either directly or indirectly attributable to substance use in Canada in 2017 (excluding tobacco) [1]. This included over 11,000 substance poisoning deaths, 99% of which were attributable to alcohol, opioids, other central nervous system (CNS) depressants (such as benzodiazepines and barbiturates), cocaine, and other CNS stimulants (such as amphetamines). Over the past decade, rates of substance poisoning deaths have increased dramatically in Canada [2, 3]. This increase has largely been associated with the non-medical use of prescription opioids or other illegal opioids [2, 4–6]. However, since 2015, evidence suggests increases in stimulant use across Canada began contributing to rising numbers of substance-related poisoning deaths [5, 7–9]. This evidence suggests co-use of opioids and stimulants as well as other patterns of polysubstance use may be contributing to the increase in substance poisoning deaths [9–12].

Polysubstance use, i.e., the concurrent or simultaneous use of more than one substance, is common in Canada [13–17]. Compared to individuals using primarily one substance, polysubstance use is more reliably associated with mental illness [18, 19], negative social and financial impacts [20], and poor treatment outcomes [21, 22]. Polysubstance use is also associated with elevated risk of fatal and non-fatal substance-related poisonings [18, 23, 24]. Despite the frequency of polysubstance use and its strong association with substance-related morbidity and mortality, there are currently no national-level statistics on the impacts of polysubstance use in Canada. To fill this gap, this study presents counts, rates and proportions of polysubstance-related poisoning deaths involving alcohol, opioids, other CNS depressants, cocaine, and other CNS stimulants excluding cocaine using data acquired from the Canadian Vital Statistics Death Database for the years 2014 to 2017.

## Methods

### Data sources and cause of death variables

All methods were performed in accordance with the relevant guidelines and regulations. This study examined death record data from the Statistics Canada holdings of the Multiple Cause of Death files (MCOD). The MCOD database provides information on a single underlying cause of death (UCD), up to 20 additional causes and demographic data from all provincial and territorial vital statistics registries on all deaths occurring in Canada. An underlying cause of death is defined as the “disease or injury which initiated the train of events leading directly to death, or the circumstances of the accident or violence which produced the fatal injury” [25]. The UCD and contributing causes of death are listed by the medical examiner/coroner and classified according to the World Health Organization “International Statistical Classification of Diseases and Related Health Problems 10^th^Revision” [25]. The MCOD files include not only the underlying cause of death, but also the immediate cause of death and other intermediate and contributory conditions listed on the death certificate. Population data used for generating crude rates were obtained from Statistics Canada [26].

### Case selection and inclusion criteria

The dataset includes all death records from January 1^st^, 2014 to December 31^st^, 2017 where a substance poisoning is listed as the UCD. A substance poisoning death is an acute toxicity death resulting from the direct effects of consuming an exogenous substance and can be intentional or unintentional[25]. ICD-10 codes for the following substance categories were included: alcohol, opioids, other central nervous system (CNS) depressants, cocaine, and CNS stimulants excluding cocaine. Poisoning deaths resulting from other substance use categories were not included in this categorical analysis; previous work has shown that virtually all substance use poisoning deaths occur within the five chosen substance use categories[27]. The analysis includes the ICD-10 codes X41-X45 (unintentional poisoning) and X61-65 (intentional poisoning). The codes for undetermined intent (Y11-Y15), were also included and considered as unintentional poisonings in this study. The multiple cause of death data using the ICD-10 “T” codes is used to identify poisoning deaths resulting from combinations of substances across the selected substance categories. Among deaths with substance poisoning as the underlying cause, the type of substance or substance category is indicated by the following ICD-10 multiple cause-of-death codes: alcohol (T51.91, T51.92, T51.94), opioids (T40.0, T40.1, T40.2, T40.3, T40.4, or T40.6); other CNS depressants (T42.3, T42.2, T42.6, T42.7), cocaine (T40.5); and CNS stimulants excluding cocaine (T43.6). Substance-related poisoning deaths were categorized as **single substance** when only one of the five substance categories was listed as a cause of death, and **polysubstance** when two or more of the five substance categories were listed. Counts across all unique combinations of substances were obtained and stratified by year, sex, and intention. Records in which a decedent had multiple substances listed upon death is only represented once for that combination.

To meet data confidentially requirements, Statistics Canada uses a disclosure method called the Laplace mechanism leading to some loss in precision of the dataset. Briefly, the Laplace mechanism adds a measure of noise to each row-level observation so as to satisfy privacy concerns in releasing Vital Statistics data. This mechanism is applied internally by the Statistics Canada Vital Statistics Team before the data is released to researchers as standard practice. The variances of the estimates were made to equal a value of two. Application of the disclosure method can also result in negative estimations when counts are very low. Following the methodology recommended by Statistics Canada, all negative counts were truncated to zero [28]. This resulted in a small positive bias in some combination counts.

### Statistical analyses

Crude mortality rates were calculated by sex and intent for the years 2014 to 2017 and were expressed as the number of deaths reported each calendar year divided by the estimates of the July 1^st^ resident population of Canada (per 100,000 persons and 95% confidence interval [CI]).

Counts and crude mortality rates for the year 2017 exclude substance poisoning deaths in the Yukon territory as this data was not available from Statistics Canada’s Vital Statistics Database at the time of data extraction (September 2019). Analyses were carried out on all unintentional and intentional poisoning deaths and stratified by type of poisoning death (single substance versus polysubstance and across combinations of substance groups). For each year*sex stratum, proportions of all poisoning deaths involving more than one substance group (“polysubstance”) were calculated overall and for each substance group. Crude mortality rates were analyzed with Joinpoint Regression version 4.8.0.1 (National Cancer Institute, Bethesda, MD) [29] to determine whether changes between 2014 and 2017 were significant [30]. Temporal trends in proportions were assessed using the Cochran-Armitage test for linear trends, using the ExcelStat data analysis add-on for Excel. A p-value of less than 0.05 was considered statistically significant for all analyses.

## Results

### Trends in mortality rates of substance poisoning deaths

**Fig. 1:**
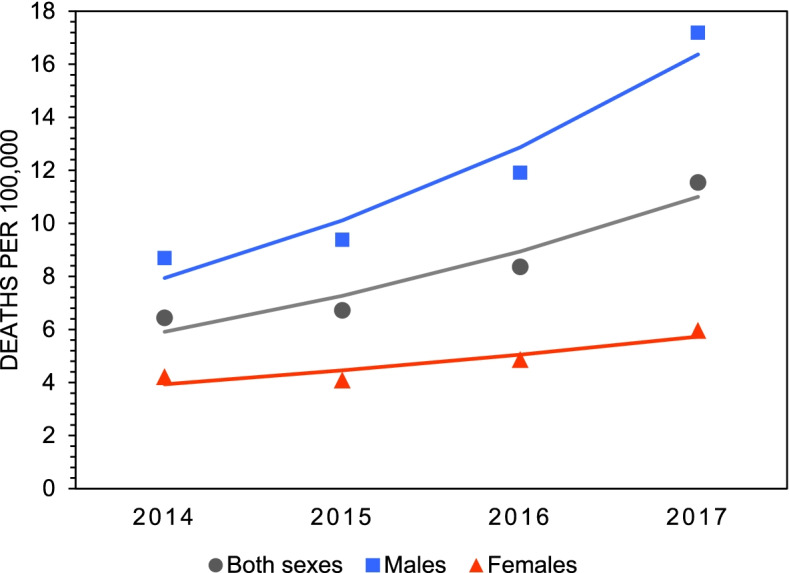
Crude mortality rates of all substance poisoning deaths by sex in Canada, 2014 - 2017

**Fig 2:**
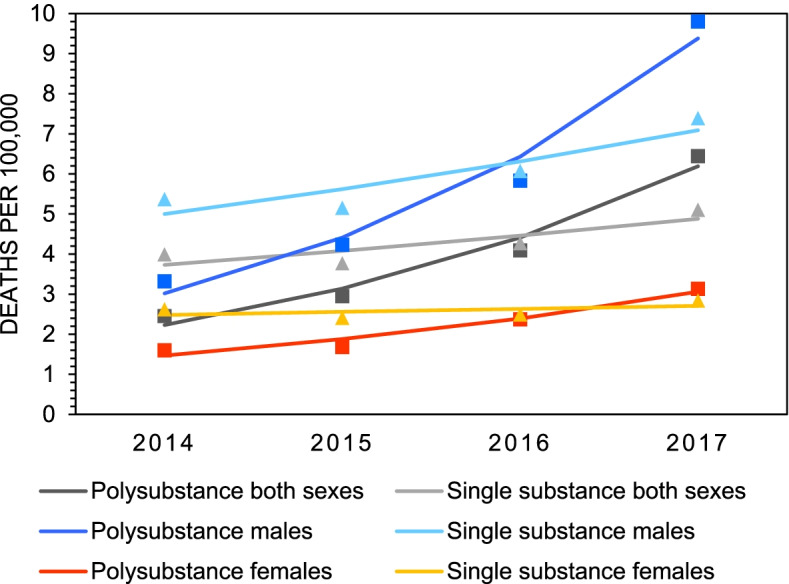
Crude mortality rates of single substance and polysubstance poisoning deaths by sex in Canada, 2014 – 2017

**Fig. 3:**
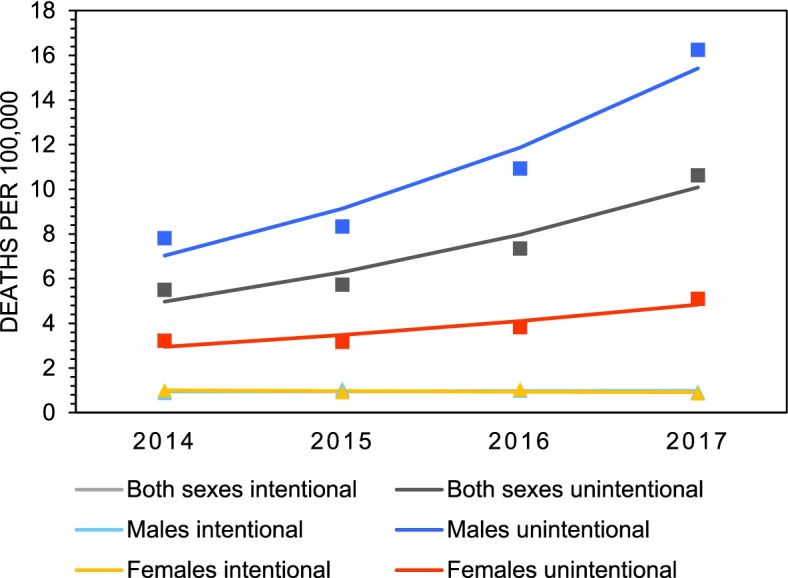
Crude mortality rates of intentional and unintentional substance poisoning deaths by sex in Canada, 2014 - 2017

Figure 1 presents the crude mortality rates of all substance poisoning deaths in Canada between 2014 and 2017. According to the data, mortality rates increased 1.8-fold among females (4.22 to 5.96 deaths/100,000) and 2.0-fold among males (8.69 to 17.19 deaths/100,000) (Table 1). In 2017, the mortality rate for substance poisoning deaths was almost three-times higher among males compared to females (Table 1). Mortality rates for polysubstance versus single substance deaths are plotted in Fig. 2 and estimates from the joinpoint analyses are presented in Table 2. The data show that mortality rates for polysubstance deaths increase significantly overall (2.6-fold, AAPC=40.5%), and among both males (3.0-fold, AAPC=45.9%) and females (2.0-fold, AAPC=27.6%). Mortality rates for deaths involving only one substance, defined as single substance deaths, remained unchanged across all groups between 2014 and 2017. Mortality rates for intentional versus unintentional deaths are plotted in Fig. 3 and estimates from the joinpoint analyses are presented in Table 3. The data show that mortality rates for unintentional deaths increase significantly overall (1.9-fold, AAPC=26.6%) and among males (2.1-fold, 29.9%). There were no significant change in rates of unintentional deaths among females. Mortality rates for intentional deaths remain unchanged across all groups (Table 3).

**Table 1:** Total counts (n), crude mortality rates^a^, 95% confidence intervals, and average annual percent change (AAPC) for substance poisoning deaths by sex in Canada, 2014 – 2017

	2014	2015	2016	2017	AAPC (%)
All poisonings					
Both sexes					
n	2281	2398	3018	4212	
Rate	6.44	6.72	8.36	11.54	23.0*
95 % CI	(6.17, 6.70)	(6.45, 6.99)	(8.06, 8.66)	(11.19, 11.89)	(2.4, 47.8)
Males					
n	1528	1662	2133	3114	
Rate	8.69	9.38	11.90	17.19	27.3*
95 % CI	(8.25, 9.13)	(8.93, 9.84)	(11.40, 12.41)	(16.59, 17.79)	(5.1, 54.1)
Females					
n	753	736	885	1098	
Rate	4.22	4.09	4.87	5.96	13.4
95 % CI	(3.92, 4.52)	(3.79, 4.39)	(4.55, 5.19)	(5.61, 6.32)	(-3.2, 32.9)

^a^ Rates are deaths per 100,000 population.

* Indicates a significant increasing trend from 2014 to 2017, *p*<0.05.

**Table 2:** Total counts (n), crude mortality rates^a^, 95% confidence intervals, and average annual percent change (AAPC) for substance poisoning deaths by sex and type (single substance versus polysubstance) in Canada, 2014 – 2017

	2014	2015	2016	2017	AAPC (%)
Polysubstance					
Both sexes					
n	868	1052	1477	2352	
Rate	2.45	2.95	4.09	6.44	40.5*
95 % CI	(2.29, 2.61)	(2.77, 3.13)	(3.88, 4.30)	(6.18, 6.70)	(17.5, 67.9)
Males					
n	583	750	1045	1776	
Rate	3.32	4.23	5.83	9.80	45.9*
95 % CI	(3.05, 3.58)	(3.93, 4.54)	(5.48, 6.19)	(9.35, 10.26)	(20.2, 77.1)
Females					
n	285	302	432	576	
Rate	1.60	1.68	2.38	3.13	27.6*
95 % CI	(1.41, 1.78)	(1.49, 1.87)	(2.15, 2.60)	(2.87, 3.38)	(7.9, 50.8)
Single substance					
Both sexes					
n	1413	1346	1541	1860	
Rate	3.99	3.77	4.27	5.10	9.3
95 % CI	(3.78, 4.19)	(3.57, 3.97)	(4.06, 4.48)	(4.86, 5.33)	(-6.5, 27.9)
Males					
n	945	912	1088	1338	
Rate	5.37	5.15	6.07	7.39	12.3
95 % CI	(5.03, 5.72)	(4.82, 5.48)	(5.71, 7.77)	(6.99, 7.78)	(-4.5, 32.1)
Females					
n	468	433	453	522	
Rate	2.62	2.41	2.49	2.84	2.9
95 % CI	(2.38, 2.86)	(2.18, 2.64)	(2.26, 2.72)	(3.08, 2.59)	(-10.7, 18.6)

^a^ Rates are deaths per 100,000 population.

* Indicates a significant increasing trend from 2014 to 2017, *p*<0.05.

**Table 3:** Total counts (n), crude mortality rates^a^, 95% confidence intervals, and average annual percent change (AAPC) for substance poisoning deaths by sex and intent (intentional versus unintentional) in Canada, 2014 – 2017

	2014	2015	2016	2017	AAPC (%)
*Unintentional*					
Both sexes					
n	1949	2047	2655	3882	
Rate	5.50	5.73	7.35	10.64	26.6*
95 % CI	(5.26, 5.74)	(5.48, 5.98)	(7.07, 7.63)	(10.30, 10.97)	(2.3, 56.8)
Males					
n	1374	1476	1958	2943	
rate	7.81	8.33	10.93	16.25	29.9*
95 % CI	(7.40, 8.22)	(7.91, 8.76)	(10.44, 11.41)	(15.66, 16.84)	(4.6, 61.3)
Females					
n	575	571	697	939	
Rate	3.22	3.18	3.83	5.10	18.0
95 % CI	(2.96, 3.48)	(2.92, 3.44)	(3.55, 4.11)	(4.77, 5.43)	(-3.2, 43.7)
*Intentional*					
Both sexes					
n	332	351	363	330	
Rate	0.94	0.98	1.01	0.90	-0.90
95 % CI	(0.84, 1.04)	(0.88, 1.09)	(0.90, 1.11)	(0.81, 1.00)	(-11.6, 11.1)
Males					
n	154	187	175	171	
Rate	0.88	1.05	0.97	0.94	1.1
95 % CI	(0.74, 1.01)	(0.90, 1.21)	(0.83, 1.12)	(0.80, 1.08)	(-15.7, 21.4)
Females					
n	178	165	189	159	
Rate	1.00	0.91	1.04	0.86	-2.8
95 % CI	(0.85, 1.14)	(0.77, 1.05)	(0.89, 1.18)	(0.73, 1.00)	(-18.5, 16.0)

^a^ Rates are deaths per 100,000 population.

* Indicates a significant increasing trend from 2014 to 2017, p < 0.05.

### Contribution of substance categories to polysubstance deaths

The percentage of unintentional substance poisoning deaths identified as polysubstance use deaths increased significantly during the study period, from 39% (756 of 1949 deaths) in 2014 to 57% (2228 of 3882 deaths) in 2017 (Table 4). To examine how each substance category (alcohol, depressants, opioids, cocaine, and other stimulants) contributed individually to total counts of unintentional polysubstance poisoning deaths, we calculated the proportion of *all* poisoning deaths for each substance where more than one substance contributed to death (i.e., “polysubstance” proportions for each substance category, Table 4). For example, among all deaths where alcohol was listed as a cause of death, this is the proportion of those records which also had another substance category present. Almost all substance categories among both males and females were increasingly likely over the study period to be involved in polysubstance deaths, as compared to single substance deaths. Indeed, in 2017 over 50% of deaths in each substance category had another substance category present. Other CNS depressants had the highest proportion of deaths that involved another substance category (95% of deaths involving other CNS depressants in 2017 included another substance category). Cocaine and other CNS stimulants also had high proportions (85% and 88% in 2017, respectively).

**Table 4:** Proportions and 95% confidence intervals of all unintentional poisoning death involving more than one substance by substance category and sex, 2014-2017

	2014	2015	2016	2017	Trend (p)^a^
*Both sexes*					
All substances	0.39 (0.37, 0.41)	0.44 (0.42, 0.47)	0.50 (0.48, 0.52)	0.57 (0.56, 0.59)	< 0.0001
Alcohol	0.49 (0.45, 0.52)	0.55 (0.51, 0.58)	0.66 (0.62, 0.69)	0.74 (0.71, 0.76)	< 0.0001
Opioids	0.51 (0.48, 0.54)	0.58 (0.55, 0.60)	0.61 (0.59, 0.63)	0.66 (0.64, 0.68)	< 0.0001
Other CNS Depressants	0.89 (0.85, 0.93)	0.93 (0.91, 0.96)	0.94 (0.92, 0.97)	0.95 (0.93, 0.97)	< 0.01
Cocaine	0.77 (0.73, 0.81)	0.77 (0.74, 0.80)	0.78 (0.76, 0.81)	0.85 (0.83, 0.86)	< 0.0001
Other CNS Stimulants*	0.71 (0.64, 0.77)	0.71 (0.65, 0.76)	0.79 (0.75, 0.82)	0.88 (0.86, 0.90)	< 0.0001
*Males*					
All substances	0.38 (0.36, 0.41)	0.45 (0.43, 0.48)	0.50 (0.48, 0.52)	0.58 (0.56, 0.60)	< 0.0001
Alcohol	0.50 (0.46, 0.54)	0.57 (0.53, 0.61)	0.68 (0.64, 0.73)	0.76 (0.73, 0.80)	< 0.0001
Opioids	0.51 (0.47, 0.54)	0.59 (0.56, 0.62)	0.62 (0.59, 0.64)	0.66 (0.64, 0.68)	< 0.0001
Other CNS Depressants	0.90 (0.85, 0.95)	0.96 (0.93, 0.99)	0.94 (0.91, 0.98)	0.97 (0.95, 0.99)	< 0.05
Cocaine	0.76 (0.71, 0.80)	0.77 (0.73, 0.81)	0.77 (0.74, 0.80)	0.85 (0.83, 0.87)	< 0.0001
Other CNS Stimulants*	0.70 (0.61, 0.78)	0.71 (0.65, 0.77)	0.78 (0.74, 0.82)	0.89 (0.87, 0.92)	< 0.0001
*Females*					
All substances	0.40 (0.40, 0.40)	0.42 (0.42, 0.42)	0.50 (0.49, 0.50)	0.55 (0.55, 0.55)	< 0.0001
Alcohol	0.45 (0.45, 0.46)	0.46 (0.45, 0.47)	0.57 (0.57, 0.58)	0.66 (0.65, 0.66)	< 0.0001
Opioids	0.52 (0.52, 0.53)	0.54 (0.54, 0.54)	0.60 (0.59, 0.60)	0.65 (0.65, 0.65)	< 0.0001
Other CNS Depressants	0.87 (0.86, 0.87)	0.90 (0.90, 0.91)	0.94 (0.93, 0.94)	0.91 (0.91, 0.92)	n.s.
Cocaine	0.80 (0.80, 0.81)	0.76 (0.76, 0.77)	0.84 (0.84, 0.85)	0.84 (0.83, 0.84)	n.s.
Other CNS Stimulants*	0.73 (0.71, 0.75)	0.68 (0.67, 0.70)	0.79 (0.79, 0.80)	0.85 (0.84, 0.85)	< 0.01

* Stimulants excluding cocaine e.g., methamphetamine

^a^ Cochran-Armitage trend test (Monte Carlo method - Number of simulations = 5000) / Two-tailed test. Significant increasing trends from 2014 to 2017 at the *p*<0.0001 level (****), *p*<0.01 level (**), and *p*<0.05 level (*)

n.s. indicates no significant trends from 2014 to 2017

### Most common polysubstance combinations

**Fig. 4:**
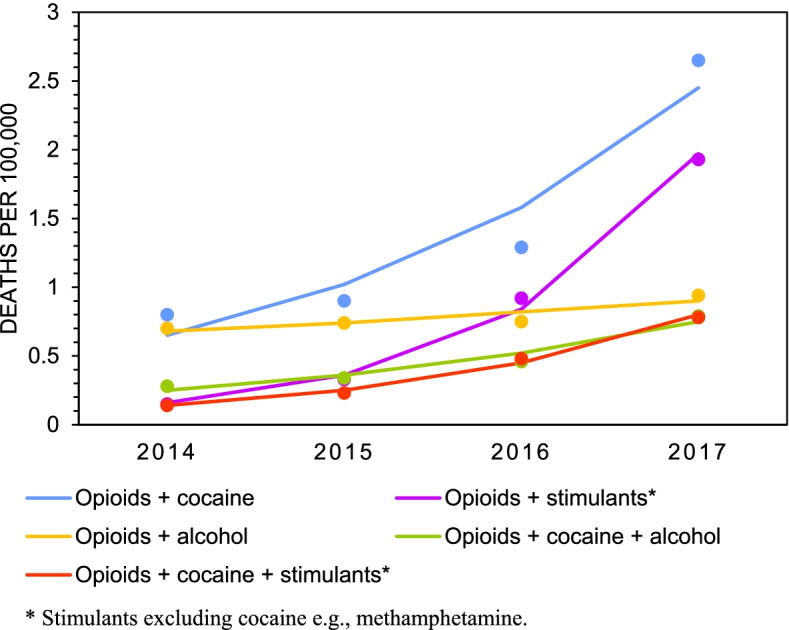
Crude death rates among males for top five most frequent combinations of unintentional poisonings involving multiple substances (“polysubstance”), 2014-2017

**Fig. 5:**
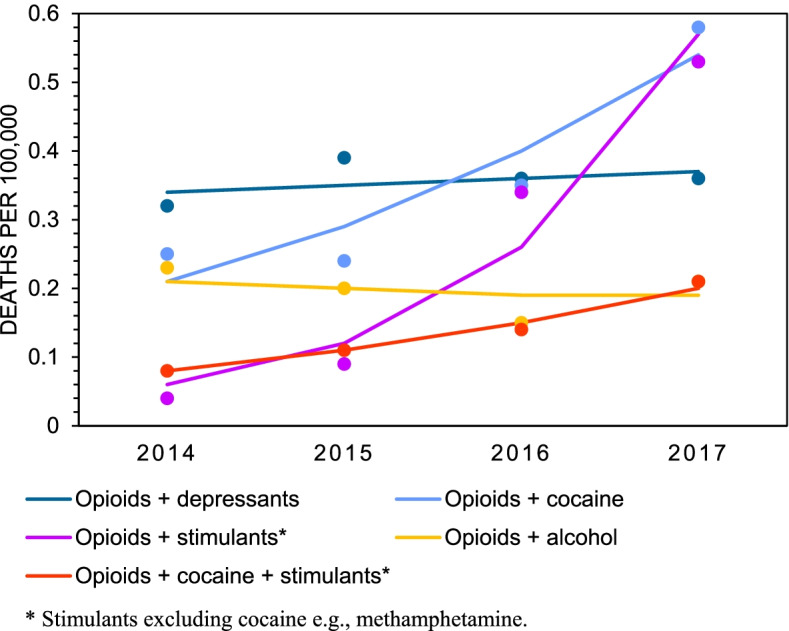
Crude death rates among females for top five most frequent combinations of unintentional poisonings involving multiple substances (“polysubstance”), 2014-2017

We next determined the five most frequent polysubstance poisonings that caused unintentional deaths between 2014 and 2017 towards understanding which substance combinations were the most likely to be involved in unintentional poisoning deaths. Mortality rates for the most frequent polysubstance combinations among males are plotted in Fig. 4, and the most frequent polysubstance combinations among females are plotted in Fig. 5. Counts, rates, and estimates from the joinpoint analyses are presented in Table 5. The data show that the largest increases in polysubstance mortality rates among males occurred from combinations of opioids and other CNS stimulants (excluding cocaine) (12.9-fold increase, AAPC=133.1%), opioids, cocaine, and other CNS stimulants (2.8-fold increase, AAPC=79.9%) and opioids and cocaine (3.3-fold increase, AAPC=55.5%). Among females, the largest increases in polysubstance mortality rates occurred from combinations of opioids and other CNS stimulants (excluding cocaine) (13.3-fold increase, AAPC=118.1%), opioids, cocaine, and other CNS stimulants (2.6-fold increase, AAPC=38.4%), and opioids and cocaine (2.3-fold increase, 37.4%). In 2017, opioids and cocaine, and opioids and CNS stimulants (excluding cocaine) were the most common polysubstance unintentional poisoning deaths among both males and females. All the top polysubstance combinations among both males and females included opioids, and mortality rates for almost all of these polysubstance combinations increased significantly between 2014 and 2017. Combinations of opioids and alcohol did not increase significantly for either males or females in this period. The same was true for combinations of opioids and other CNS depressants for females.

**Table 5:** Total counts (n), crude mortality rates^a^, 95% confidence intervals, and average annual percent change (AAPC) for unintentional polysubstance poisoning deaths by sex, 2014 – 2017

	2014	2015	2016	2017	AAPC (%)
*Males*					
Opioids + Cocaine					
n	140	160	232	480	
Rate	0.80	0.90	1.29	2.65	55.5*
95 % CI	(0.67, 0.93)	(0.76, 1.04)	(1.13, 1.46)	(2.41, 2.89)	(5.3, 129.4)
Opioids + Other CNS Stimulants^b^					
n	26	58	166	349	
Rate	0.15	0.33	0.92	1.93	133.1**
95 % CI	(0.09, 0.21)	(0.24, 0.41)	(0.78, 1.06)	(1.72, 2.13)	(87.7, 189.6)
Opioids + Alcohol					
n	122	132	135	169	
Rate	0.70	0.74	0.75	0.94	9.9
95 % CI	(0.57, 0.82)	(0.62, 0.87)	(0.63, 0.88)	(0.79, 1.08)	(-3.5, 25.0)
Opioids + Cocaine + Other CNS Stimulants^b^					
n	24	41	86	142	
Rate	0.14	0.23	0.48	0.78	79.9*
95 % CI	(0.08, 0.19)	(0.16, 0.30)	(0.38, 0.58)	(0.65, 0.91)	(53.4, 110.9)
Opioids + Cocaine + Alcohol					
n	49	60	82	143	
Rate	0.28	0.34	0.46	0.79	44.4*
95 % CI	(0.20, 0.36)	(0.25, 0.43)	(0.36, 0.56)	(0.66, 0.92)	(13.3, 83.8)
*Females*					
Opioids + Cocaine					
n	44	44	64	107	
Rate	0.25	0.24	0.35	0.58	37.4
95 % CI	(0.17, 0.32)	(0.17, 0.32)	(0.27, 0.44)	(0.47, 0.69)	(-0.6, 90.1)
Opioids + Other CNS Stimulants^b^					
n	8	16	61	97	
Rate	0.04	0.09	0.34	0.53	118.1*
95 % CI	-	(0.04, 0.13)	(0.25, 0.42)	(0.42, 0.63)	(10.2, 331.7)
Opioids + Other CNS Depressants					
n	57	70	66	67	
Rate	0.32	0.39	0.37	0.37	2.9
95 % CI	(0.24, 0.40)	(0.30, 0.48)	(0.28, 0.45)	(0.28, 0.45)	(-13.4, 22.3)
Opioids + Alcohol					
n	41	36	27	39	
Rate	0.23	0.20	0.15	0.21	-4.6
95 % CI	(0.16, 0.30)	(0.14, 0.27)	(0.09, 0.21)	(0.15, 0.28)	(-34.1, 38.1)
Opioids + Cocaine + Other CNS Stimulants^b^					
n	14	19	25	38	
Rate	0.08	0.11	0.14	0.21	38.4**
95 % CI	(0.04, 0.12)	(0.06, 0.15)	(0.08, 0.19)	(0.14, 0.27)	(25.3, 52.8)

^a^ Rates are deaths per 100,000 population

^b^ Stimulants excluding cocaine e.g., methamphetamine

“-“ indicates that the 95% CI is not reliable enough to report

* Indicates a significant increasing trend from 2014 to 2017, *p*<0.05

** Indicates a significant increasing trend from 2014 to 2017, *p*<0.01

### Interpretation

We examined the contribution of polysubstance use to substance poisoning deaths in Canada between 2014 and 2017. We show that increasing rates of unintentional poisoning deaths in Canada during this time were largely attributable to increasing rates of polysubstance poisoning deaths. Polysubstance poisoning mortality rates increased far more than single-substance death rates, and by 2017, more than half of all unintentional poisoning deaths were polysubstance deaths. These increases were much more dramatic among males. All substance categories examined during the study period were increasingly involved in polysubstance deaths, as compared to single substance deaths. Combinations of opioids with cocaine and/or CNS stimulants excluding cocaine were associated with the largest increases in unintentional polysubstance poisoning deaths during the study and accounted for the most unintentional deaths from polysubstance use in 2017.

Canadian data indicate that unintentional poisoning deaths have increased almost 3-fold between 2008 and 2018 in Canada [3]. Rates of unintentional poisoning deaths in Canada have further accelerated since the start of the COVID-19 pandemic [4, 9, 31]. This increase has been largely driven by the use (either deliberate or accidental) of synthetic opioids like fentanyl [2, 4, 6, 9]. According to toxicology data in both Canadian and American jurisdictions, trends in overdose deaths involving cocaine and other stimulants have also increased substantially in the past five to ten years [4–6, 32, 33]. Synthetic opioids, mostly illegally sourced fentanyl, also underlie these recent increases in stimulant-related poisoning deaths [2, 5, 9, 10, 34–36]. This is likely driven in part by consuming drugs from the illegal supply that contain both synthetic opioids and stimulants (whether intentionally or not). Toxicological analysis of illegal drugs seized by Canadian law enforcement agencies suggests that, when stimulants do co-occur with opioids in the same drug sample, the opioid is usually fentanyl or one of its analogues [8]. Data from the United States further demonstrate that opioid-related overdoses in recent years are generally more likely to involve at least one other substance category [36–41]. Hence, polysubstance use, and more specially the co-use of opioids and stimulants, is becoming a primary driver of increased substance poisoning deaths in Canada.

Results suggest the use of opioids in combination with other substances, particularly stimulants, needs to be addressed in efforts to curb the epidemic of opioid deaths in Canada across the spectrum of prevention, harm reduction, and treatment. Specifically, measures that address the unpredictability of the illegal drug supply would reduce harms related to polysubstance use (whether multiple substances are used deliberately or not) [8]. These may include expanding access to reliable information about drug contents through drug checking services, as well as access to overdose prevention services such as supervised consumption/ overdose prevention sites and take home naloxone kits [42]. Drug contamination and other upstream risk factors may be addressed by interventions that provide a legal, pharmaceutical-grade supply of opioids and non-opioid substances [43–45]. In all cases, people who use drugs must be involved throughout implementation and evaluation to ensure services are relevant to the preferences and needs of people who use drugs in different contexts [45–47].

In general, prevention efforts can target common pathways that lead to substance use disorders, treatment strategies can focus on common features across substances, and service delivery can also be structured in a way to address issues associated with multiple types of substance use [23, 37, 41]. Polysubstance use also has important implications for public health surveillance and research. Identifying factors (e.g., demographics, behaviors) linked to polysubstance use can inform interventions and prevention pathways, while patterns of polysubstance use should be monitored alongside trends of other substances in a timely fashion, particularly as the COVID-19 pandemic has rapidly impacted the illegal drug supply and substance use behaviors.

### Limitations

We limited our analysis to death records for poisoning deaths involving only five different substance categories that frequently cause substance poisoning deaths [3, 46–48]. Cannabis, hallucinogens, solvents and inhalants, antidepressants and other psychoactive substances were excluded from the analysis. Methodologically, this exclusion was necessary to avoid very low counts (close to zero or negative estimations) for polysubstance combinations that appeared infrequently in the vital statistics database. Although there is evidence suggesting that these other substance classes more rarely cause fatal poisonings on their own [5, 49–51], they could be involved in poisoning deaths primarily caused by other substances [5, 52–54] and warrant further investigation to better characterize the polysubstance nature of poisoning deaths. In addition, our definition of polysubstance is conservative and confined by broad substance categories; the dataset analysed for this study does not differentiate between different types of substances within each category. For instance, a poisoning death caused by both heroin and fentanyl for example, would be considered a single substance poisoning death.

Our study methodology also does not distinguish between deliberate polysubstance use (when an individual explicitly uses two or more substances), and accidental polysubstance use (when an individual thinks they are using only one substance, but it is contaminated or adulterated by another substance category). Both deliberate and accidental polysubstance use may underlie increases in unintentional poisoning deaths from the co-use of opioids and stimulants. For instance, fentanyl and related synthetic opioids are often consumed unknowingly with other substances [8, 34, 55–58] while the popularity and desire for using both opioids and stimulants has also grown among people who use either substance [56, 59]. These different pathways leading to polysubstance use is an area for further investigation.

Another important limitation of this dataset is that counts of poisoning deaths are not segregated by age, as this would result in small estimates that would need to be supressed to prevent identification of individual records. Therefore, we were unable to investigate any age-specific trends or calculate any age-adjusted death rates. An analysis of demographic characteristics from a comparable dataset from overall intentional and unintentional injuries for 2017, which are largely (but not exclusively) accounted for by substance use poisonings, shows the majority of intentional and unintentional injury deaths occurred in the 35-64 age group (See Supplementary Table 1). Finally, our study ultimately depends on the accuracy of toxicology screenings carried out by coroners and medical examiners, and proper identification and coding of substances as contributing causes of death. Not only can these processes be imperfect, but there can be inconsistent practices in toxicology screening and testing leading to inaccuracies in death records for substance poisoning deaths [2, 60, 61]. More specifically, because collecting evidence to ascertain intent is especially difficult, intentional deaths (suicides) may frequently be misclassified as unintentional deaths or deaths with undetermined intent [61]. This would lead to an overestimation of the number and rates of unintentional poisoning deaths in our study. The time required to complete death investigations and classify the cause of death may have also resulted in an underestimation of poisoning deaths in our study, particularly for more recent years.

## Conclusion

To our knowledge, this is the first study examining national patterns of polysubstance poisoning deaths in the era of the opioid epidemic. A multitude of intersecting factors are causing increased rates of substance poisoning deaths in Canada, including increased use of stimulants from the illegal drug supply, contamination of the illegal drug supply with fentanyl/synthetic opioids, and a changing landscape of polysubstance use where co-consumption of opioids, stimulants and other substances is increasingly more popular. Improved surveillance of polysubstance use patterns and harms will be essential to informing responses to the ongoing opioid crisis in Canada. As the COVID-19 pandemic continues to fuel substance poisoning deaths, these data highlight the need for essential services and supports to be accessible for people most at risk and the need to expand prevention, treatment, and harm reduction activities.

## Supplementary Information

**Additional file 1: Table 1.** Total counts (n) and percentage (%) of substance use-attributable injury deaths in Canada by region, age group and sex, 2017.

## Data Availability

The results/data/figures in this manuscript have not been published elsewhere, nor are they under consideration by another publisher. The authors are not able to share the raw data. The dataset used to conduct this analysis was acquired through Statistics Canada and is not owned by the authors. Due to the data sharing agreement the authors established with Statistics Canada, we are unable to share the raw data publicly, however the data used for this study can be acquired directly from Statistics Canada.

## Abbreviations

AAPC: Average Annual Percent Change
CI: Confidence Interval
CNS: Central Nervous System
CVSD: Canadian Vital Statistics Death Database
MCOD: Multiple Cause of Death
UCD: Underlying Cause of Death
