# Supplementary material for: Polysubstance use poisoning deaths in Canada: an analysis of trends from 2014 to 2017 using mortality data
Source: BMC Public Health. 2022 Feb 10;22:269. doi: 10.1186/s12889-022-12678-z (PMC8830122; doi:10.1186/s12889-022-12678-z)
Supplement: Supplementary file 1 — Additional file 1: Table 1. Total counts (n) and percentage (%) of substance use-attributable injury deaths in Canada by region, age group and sex, 2017. [file 12889_2022_12678_MOESM1_ESM.docx]

**Supplementary material**

**Table 1. Total counts (n) and percentage (%) of substance use-attributable injury deaths in Canada by region, age group and sex, 2017.**

|  |  | Intentional injury | | | Unintentional injury | | | Total | | |
| --- | --- | --- | --- | --- | --- | --- | --- | --- | --- | --- |
| *Region* |  | All substances^1^ | Opioids | Other CNS Stimulants^2^ | All substances^1^ | Opioids | Other CNS Stimulants^2^ | All substances^1^ | Opioids | Other CNS Stimulants^2^ |
| Newfoundland and Labrador | n | 102.41 | 35.77 | 8.89 | 68.64 | 22.98 | 0.77 | 171.05 | 58.74 | 9.66 |
|  | % | 2.36 | 2.12 | 3.94 | 1.02 | 0.72 | 1.69 | 1.54 | 1.20 | 3.56 |
| Prince Edward Island | n | 36.60 | 9.86 | 4.02 | 22.44 | 12.60 | 0.38 | 59.04 | 22.46 | 4.41 |
|  | % | 0.84 | 0.58 | 1.78 | 0.33 | 0.39 | 0.84 | 0.53 | 0.46 | 1.63 |
| Nova Scotia | n | 148.84 | 57.32 | 11.71 | 144.93 | 48.96 | 1.79 | 293.77 | 106.28 | 13.50 |
|  | % | 3.43 | 3.40 | 5.19 | 2.15 | 1.53 | 3.93 | 2.65 | 2.17 | 4.98 |
| New Brunswick | n | 90.52 | 35.73 | 4.74 | 96.74 | 29.71 | 1.65 | 187.25 | 65.44 | 6.39 |
|  | % | 2.09 | 2.12 | 2.10 | 1.44 | 0.93 | 3.61 | 1.69 | 1.34 | 2.36 |
| Quebec | n | 825.50 | 278.11 | 48.95 | 639.03 | 199.64 | 3.20 | 1464.53 | 477.75 | 52.15 |
|  | % | 19.04 | 16.50 | 21.70 | 9.48 | 6.23 | 7.02 | 13.22 | 9.77 | 19.24 |
| Ontario | n | 1468.63 | 600.38 | 71.77 | 2442.50 | 1086.01 | 15.53 | 3911.13 | 1686.39 | 87.31 |
|  | % | 33.87 | 35.62 | 31.82 | 36.24 | 33.89 | 34.10 | 35.31 | 34.49 | 32.20 |
| Manitoba | n | 228.69 | 85.63 | 10.14 | 257.42 | 93.11 | 2.70 | 486.11 | 178.74 | 12.84 |
|  | % | 5.27 | 5.08 | 4.50 | 3.82 | 2.91 | 5.92 | 4.39 | 3.66 | 4.74 |
| Saskatchewan | n | 187.62 | 70.11 | 4.71 | 179.18 | 69.61 | 1.27 | 366.80 | 139.72 | 5.97 |
|  | % | 4.33 | 4.16 | 2.09 | 2.66 | 2.17 | 2.78 | 3.31 | 2.86 | 2.20 |
| Alberta | n | 670.40 | 267.58 | 33.37 | 1157.57 | 612.49 | 8.37 | 1827.97 | 880.07 | 41.74 |
|  | % | 15.46 | 15.87 | 14.80 | 17.17 | 19.11 | 18.37 | 16.50 | 18.00 | 15.40 |
| British Columbia | n | 512.51 | 222.40 | 22.07 | 1706.63 | 1023.12 | 9.75 | 2219.15 | 1245.52 | 31.82 |
|  | % | 11.82 | 13.19 | 9.78 | 25.32 | 31.93 | 21.41 | 20.03 | 25.47 | 11.74 |
| Yukon | n | - | - | - | - | - | - | - | - | - |
|  | % | - | - | - | - | - | - | - | - | - |
| Northwest Territories | n | 23.57 | 6.65 | 1.86 | 12.66 | 2.99 | 0.04 | 36.23 | 9.64 | 1.91 |
|  | % | 0.54 | 0.39 | 0.83 | 0.19 | 0.09 | 0.10 | 0.33 | 0.20 | 0.70 |
| Nunavut | n | 41.08 | 16.07 | 3.31 | 12.26 | 3.36 | 0.10 | 53.34 | 19.43 | 3.42 |
|  | % | 0.95 | 0.95 | 1.47 | 0.18 | 0.10 | 0.23 | 0.48 | 0.40 | 1.26 |
| Canada | n | 4336.38 | 1685.60 | 225.56 | 6740.00 | 3204.59 | 45.55 | 11076.38 | 4890.18 | 271.11 |
|  | % | 100.00 | 100.00 | 100.00 | 100.00 | 100.00 | 100.00 | 100.00 | 100.00 | 100.00 |
| *Age Group* |  | All substances^1^ | Opioids | Other CNS Stimulants^2^ | All substances^1^ | Opioids | Other CNS Stimulants^2^ | All substances^1^ | Opioids | Other CNS Stimulants^2^ |
| 0-14 | n | 28.41 | 7.90 | 8.31 | 36.78 | 14.70 | 0.44 | 65.19 | 22.60 | 8.75 |
|  | % | 0.66 | 0.47 | 3.69 | 0.55 | 0.46 | 0.96 | 0.59 | 0.46 | 3.23 |
| 15-34 | n | 1543.26 | 500.02 | 33.57 | 2001.31 | 1124.56 | 15.17 | 3544.57 | 1624.57 | 48.74 |
|  | % | 35.59 | 29.66 | 14.88 | 29.69 | 35.09 | 33.30 | 32.00 | 33.22 | 17.98 |
| 35-64 | n | 2162.70 | 910.26 | 135.18 | 3502.10 | 1933.34 | 26.72 | 5664.81 | 2843.60 | 161.90 |
|  | % | 49.87 | 54.00 | 59.93 | 51.96 | 60.33 | 58.65 | 51.14 | 58.15 | 59.72 |
| 65+ | n | 602.01 | 267.42 | 48.49 | 1199.81 | 131.99 | 3.23 | 1801.81 | 399.41 | 51.72 |
|  | % | 13.88 | 15.86 | 21.50 | 17.80 | 4.12 | 7.08 | 16.27 | 8.17 | 19.08 |
| Total | n | 4336.38 | 1685.60 | 225.56 | 6740.00 | 3204.59 | 45.55 | 11076.38 | 4890.18 | 271.11 |
|  | % | 100.00 | 100.00 | 100.00 | 100.00 | 100.00 | 100.00 | 100.00 | 100.00 | 100.00 |
| *Sex* |  | All substances^1^ | Opioids | Other CNS Stimulants^2^ | All substances^1^ | Opioids | Other CNS Stimulants^2^ | All substances^1^ | Opioids | Other CNS Stimulants^2^ |
| Females | n | 1044.50 | 483.57 | 103.91 | 1760.16 | 777.90 | 31.61 | 2804.66 | 1261.47 | 135.51 |
|  | % | 24.09 | 28.69 | 46.07 | 26.12 | 24.27 | 69.39 | 25.32 | 25.80 | 49.99 |
| Males | n | 3291.88 | 1202.03 | 121.65 | 4979.84 | 2426.69 | 13.94 | 8271.72 | 3628.72 | 135.59 |
|  | % | 75.91 | 71.31 | 53.93 | 73.88 | 75.73 | 30.61 | 74.68 | 74.20 | 50.01 |
| Total | n | 4336.38 | 1685.60 | 225.56 | 6740.00 | 3204.59 | 45.55 | 11076.38 | 4890.18 | 271.11 |
|  | % | 100.00 | 100.00 | 100.00 | 100.00 | 100.00 | 100.00 | 100.00 | 100.00 | 100.00 |

^1 Alcohol, cocaine, other CNS stimulants, opioids and other CNS depressants.^

^2 Stimulants excluding cocaine (e.g., methamphetamine).^

^“-” indicates that data on injury deaths in Yukon were not available for year 2017.^

^Note: Intentional and unintentional injury deaths consist primarily of substance use poisoning deaths, but also include deaths due to assault/homicide and other intentional self-harm (for alcohol, cocaine, other CNS stimulants, opioids and other CNS depressants) and falls, drowning, fires, other unintentional injuries, accidental poisoning by substances other than alcohol, other intentional injuries and intentional self-poisoning by substances other than alcohol (for alcohol only). Negative counts were rounded to zero. Data retrieved from https://csuch.ca/explore-the-data/.^
